# Transcatheter arterial chemoembolization combined with Hippo/YAP inhibition significantly improve the survival of rats with transplanted hepatocellular carcinoma

**DOI:** 10.1186/s12944-021-01486-w

**Published:** 2021-07-25

**Authors:** Yi Quan, Zhi Li, Kangshun Zhu, Jundi Liang

**Affiliations:** 1 Department of Oncology Medilcal Center, The First People’s Hospital of Zhaoqing, Zhaoqing, Guangdong 526000 China; 2 Department of Interventional, First Affiliated Hospital of Suzhou University, Suzhou, Jiangsu 215006 China; 3 Department of Minimally Invasive Medicine, Second Affiliated Hospital of Guangzhou Medical University, Guangzhou, 510000 China

**Keywords:** Transplanted hepatocellular carcinoma, Transcatheter arterial chemoembolization, Hippo/YAP signaling pathway, Verteporfin, Prognosis

## Abstract

**Background:**

This study aimed to explore the effect of inhibiting the Hippo/Yes-associated protein (YAP) signaling pathway on the outcomes of transcatheter arterial chemoembolization (TACE) in treating transplanted hepatocellular carcinoma (HCC).

**Methods:**

A transplanted HCC rat model was established. Then, rats were randomly divided into four groups: Sham, TACE, verteporfin (inhibitor of Hippo/YAP), and TACE+verteporfin. Lent-OE-YAP was transfected into rats to overexpress YAP in vivo. After treatments, morphological changes, tumor weight, and the overall survival of rats in different groups were analyzed. Real-time PCR, immunohistochemistry staining, and Western blotting were used to determine the expression of factors related to the Hippo/YAP signaling pathway.

**Results:**

Tumor weight and tissue lesions in the TACE and verteporfin groups were significantly reduced compared with the Sham group. Verteporfin significantly decreased tumor weight after TACE treatment. In addition, verteporfin significantly improved the overall survival of rats with transplanted HCC after TACE treatment. Compared with the Sham group, both TACE and verteporfin groups exhibited significantly decreased expression of macrophage-stimulating (MST)1, MST2, long-acting thyroid stimulator 1, transcriptional co-activator with PDZ-binding motif (TAZ), Yes-associated protein (YAP), TEA domain transcription factor (TEAD)1, TEAD2, TEAD3, and TEAD4. TACE plus verteporfin significantly enhanced the downregulation of effectors in the Hippo/YAP signaling pathway and decreased tumor size, while the overexpression of YAP exerted opposite effects.

**Conclusion:**

The inhibition of the Hippo/YAP signaling pathway via verteporfin significantly improved the outcomes of TACE in treating transplanted HCC.

**Supplementary Information:**

The online version contains supplementary material available at 10.1186/s12944-021-01486-w.

## Introduction

Hepatocellular carcinoma (HCC) is one of the most common malignancies worldwide and approximate 70% of the cases occur in Asian countries [1]. Many risk factors contribute to the development of HCC in humans, such as virus infection, chemicals, alcohol, and genetic factors [2, 3]. Hepatitis B and C virus (HBV and HCV) are two major risk factors for HCC [4, 5]. During HBV and HCV infection, environmental and genetic factors may promote the development of HCC [6, 7]. Despite multiple efforts that have been made, the pathological mechanisms of HCC remain unknown and need to be further elucidated.

The Hippo/Yes-associated protein (YAP) signaling pathway is commonly involved in the regulation of cell proliferation, differentiation, and metabolism [8, 9]. The Hippo/YAP signaling pathway plays an essential role in HCC. The Hippo pathway is a suppressor that has been shown to inhibit cell growth when it is activated. However, gene fusion of its downstream effectors can lead to neoplasia by promoting cell proliferation, and decreasing apoptosis and differentiation [10]. YAP, as a pivotal downstream effector of the Hippo signaling pathway, functions mainly via the TEA domain (TEAD) family transcription factors to activate gene expression [11]. The transcriptional coactivator with PDZ binding motif (TAZ) is regulated by the Hippo pathway and is paralogous with YAP [12]. Zhang et al. showed that transforming growth factor beta 1 inhibited the proliferation of HCC tumor cells via targeting the Hippo signaling [13]. Fitamant et al. have found that YAP inhibition restored the differentiation of advanced HCC and thus regulated the development of tumor regression [14]. The Hippo/YAP signaling also interacts with the Notch and Wnt/β-catenin signaling pathways to repress the development of HCC [15]. In addition, the Hippo/YAP/TAZ signaling pathway regulates the pathogenesis of hepatic fibrosis to promote the formation of HCC [16]. However, the potential application of the Hippo/YAP signaling in clinics has not been reported.

Many efforts have been made to improve the prognosis of HCC and the main therapies include surgical resection, liver transplantation, chemotherapy, and locoregional treatment [17]. Transcatheter arterial chemoembolization (TACE) is a common treatment strategy for patients with unresectable HCC and often provides satisfying outcomes [18, 19]. Even though some retrospective studies have suggested that TACE treatment after refractoriness may cause liver function deterioration [20], there is no consensus on the refractoriness and unsuitability of TACE on treating HCC. Some studies have elicited that the effects of combined sorafenib with TACE for patients with HCC were uncertain [21, 22]. Hence, it is important to improve the application of TACE in treating HCC. In 2017, a research team reported that the inhibition of the Wnt/β-catenin signaling pathway significantly improved the therapeutic outcomes in TACE via attenuating the migration and invasion, and accelerating the apoptosis of tumor cells in rats with transplanted HCC [13]. Verteporfin, an FDA-approved drug blocking YAP-TEAD interaction and inhibiting YAP transcription [23], is commonly used as a pharmacological inhibitor. Verteporfin in combination with chemotherapeutic agents could overcome side effects caused by anti-cancer drugs in the treatment of HCC [24]. Zhao et al. found that evodiamine inhibited cell proliferation and facilitate apoptosis via the Hippo/YAP signaling pathway in HCC [25]. Chai et al. reported that cucurbitacin B inhibited the expression of proteins related to the Hippo/YAP signaling pathway to inhibit proliferation and induce apoptosis [26]. However, the effect of inhibiting the Hippo/YAP signaling pathway in TACE-treated transplanted HCC has not been reported.

This study aimed to explore the effect of the Hippo/YAP signaling pathway on the prognosis of TACE in treating transplanted HCC. It provided new information for improving the outcomes of TACE and using verteporfin to treat transplanted HCC.

## Materials and methods

### Construction of transplanted HCC model

This study was approved by the animal ethical standard. Forty-two Sprague-Dawlay (SD) rats, weighing 90 ± 10 g (5 weeks old), were used in this study (Shanghai Institute of Pharmaceutical Industry, Shanghai, China). Rats were kept under a specific pathogen free (SPF) condition (24–26 °C, 45–55% humidity) with free access to water. Walker-256 tumors (Shanghai Institute of Pharmaceutical Industry, Shanghai, China) were used to conduct the HCC model. Subsequently, two rats were intraperitoneally injecting with 5 × 10^5^ cells. One week later, rats were sacrificed. Ascites tumor, after formed, was extracted to obtain 800 g of ascites. Then ascites was centrifuged and washed with physiological saline. After that, the mushy cytoplasm was collected for further use. One hundred male SD rats weighed approximately 225 ± 25 g was purchased from SIPI (Shanghai, China) and a transplanted HCC model was constructed as previously reported [27]. Ten days later, the tumor of liver lobe (1 cm in diameter) in rat was observed, indicating successful establishment of the model.

### Treatment for transplanted HCC model

A total of 100 transplanted HCC model rats were randomly divided into four groups: the sham group (*n* = 25), the TACE group (*n* = 25), the verteporfin group (*n* = 25), and the TACE+verteporfin group (*n* = 25). Rats in the sham group underwent laparotomy without any other surgery. Rats in the TACE and TACE+verteporfin groups received gradual incision of the abdominal cavity by TACE. Rats in the verteporfin group were treated with 10 mg/kg verteporfin (Selleck Chemicals, Houston, USA; verteporfin was dissolved in 20 μl of dimethyl sulfoxide and diluted to 5% using saline to reduce its toxicity). Rats in the TACE+verteporfin group received verteporfin during TACE therapy. The hepatic artery and gastroduodenal artery of the model rats were removed during the whole course of treatment, and microcatheter was inserted through gastroduodenal artery. Then, 10 mg/kg verteporfin was injected into the rats for verteporfin treatment. All treatments lasted for 10 days. After that, in each group, 5 rats were sacrificed for pathological analysis and subsequent experiments and the rest rats were used to record the survival condition. To evaluate the effects of dimethyl sulfoxide, a vehicle group in which rats were treated with dimethyl sulfoxide diluted in 5% saline was set. To investigate the molecular mechanism, vectors overexpressing YAP was constructed by HanBio Co. Ltd., Shanghai, China using the lentivirus expressing system. The lent-OE-YAP was transfected into rats via tail vein injection, while lent-OE-NC was used as the control.

### Hematoxylin & Eosin (H&E) staining

After treatment, liver tumors of each group were harvested and weighed. Then, part of liver tumor tissues was fixed with 4% paraformaldehyde (PFA) (Thermo Fisher Scientific, Shanghai, China), dehydrated by gradient ethanol (Sigma, Shanghai, China) and embedded by paraffin. Subsequently, tissues were cut into 5-μm section, dewaxed using xylene (Sigma, Shanghai, China), rehydrated with gradient ethanol (Sigma, Shanghai, China), and stained with hematoxylin and eosin (Nanjing Jiancheng, Nanjing, Jiangsu, China) according to manufacturer’s protocols. Then, the pathological features of liver tumor tissues were analyzed under the light microscope.

### Immunohistochemistry (IHC)

Paraffin-embedded slides of HCC tissue were heated at 60 °C for 1 h. Slides hydrated with xylene (Sigma-Aldrich, St. Louis, MO, USA) and gradient alcohol (Sigma-Aldrich, St. Louis, MO, USA) were dewaxed. For antigen extraction, slides were put into diluted potassium citrate (Sigma, Shanghai, China) solution and microwave- heated at 90 °C for 10 min. After cooling at room temperature, slides were rinsed with phosphate buffered saline (Mechanistic) (Sigma-Aldrich, St. Louis, MO, USA) for 3 times (5 min each time). Then, 3% H_2_O_2_ (Sigma-Aldrich, St. Louis, MO, USA) were added at room temperature. Subsequently, slides were blocked with 5% goat serum (Sigma-Aldrich, St. Louis, MO, USA) at room temperature for 20 ~ 30 min, followed by incubation with primary antibodies (MST, MST2, LATS1, LATS2, YAP1, TAZ, TEAD1, TEAD2, TEDA3, TEAD4, GAPDH) at 4 °C overnight. All antibodies were rabbit resource, diluted at 1:200, and purchased from Abcam (Cambridge, MA, USA). After washed with PBS (Sigma-Aldrich, St. Louis, MO, USA) for three times, slices were incubated with a goat-anti rabbit secondary antibody (Thermo Fisher Scientific, Shanghai, China) at room temperature for 1 h and then visualized using the DAB method (ZSGB-BIO, Beijing, China) according to manufacturer’s protocol. Subsequently, slices were stained with hematoxylin (Nanjing Jiancheng, Nanjing, Jiangsu, China) according to manufacturer’s protocols to visualize nuclei. For each sample, three fields of view were selected at random. Positive cells were counted.

### Quantitative real time PCR (RT-qPCR)

After treatment, TRIzol reagent (Takara, Dalian, Shenyang, China) was utilized to extract total RNA according to manufacturer’s protocol and a cDNA synthesis kit (Tiangen, Beijing, China) was utilized to synthesize cDNA according to manufacturer’s instruction. The expressions of MST, MST2, LATS1, LATS2, YAP1, TAZ, TEAD1, TEAD2, TEDA3, TEAD4, and GAPDH (Abcam, Cambridge, MA, USA) were determined using SYBR Green Master Mix (Vazyme Biotech Co., Ltd., Nanjing, China) on a BioRad CFX96 Sequence Detection System (BioRad company, Berkeley. CA, USA). The primers (synthesized by Bo shang Biotechnology Co., Ltd., Shanghai, China) used for reaction are shown in Table 1. GAPDH was used as the internal control. The 2^-ΔΔCt^ method was used to detect the relative expression of linc01014. The premier sequences were purchased from RiBio (Guangzhou, China).

**Table 1:** The primer sequences for RT-qPCR

Gene type	Primer sequence
**MST**	Forward: 5′-CCTCCCACATTCCGAAAACCA-3′
	Reverse: 5′-GCACTCCTGACAAATGGGTG-3′
**MST2**	Forward: 5′-AGGAACA-GCAACGAGAATTGG-3′
	Reverse: 5′-CCCCTTCACTCATCGTGCTT-3′
**LATS1**	Forward: 5′-AATTTGGGACGCATCATAAAGCC-3′
	Reverse: 5′-TCGTCGAGGATCTTGGTAACTC-3′
**LATS2**	Forward: 5′-GCTTCATCCACCGAGACATCAA-3′
	Reverse: 5′-CGACAGTTAGACACATCATCCCAGA-3′
**YAP1**	Forward: 5′-CCTGATGGATGGGAACAAGC-3′
	Reverse:: 5′-GCACTCTGACTGATTCTCTGG-3′
**TAZ**	Forward: 5′-ACGTCCTTCCTAACAGTCC-3′
	Reverse: 5′-TGCCTGACTCTTCAGATGC-3′
**TEAD1**	Forward: 5′-CGCCTTCTTCCTCGTCAA-3′
	Reverse: 5′-TCGCATACTCCGTCTCTAC-3′
**TEAD2**	Forward: 5′-CCACATGCCTTCTTCCTCGTCAA-3′
	Reverse: 5′-CCGTCTCCACCTTCTCTACCACTT-3′
**TEAD3**	Forward: 5′-TCGGCAAGCAGGTGGTAGAGAAG-3′
	Reverse: 5′-CAGGCAGGTGTGTGGAGGATGT-3′
**TEAD4**	Forward: 5′-CTGACGGAGGAAGGCAAGATGTATG-3′
	Reverse: 5′-ACGGGCAAGCACCTGGATGT-3′
**GAPDH**	Forward: 5′-CCTTCCGTGTTCCTACCCC-3′
	Reverse: 5′-GCCCAAGATGCCCTTCAGT-3′

### Western blotting

A total of 0.05 μg tissue samples were collected using RIPA lysis buffer (Millipore, Billerica, MA, USA) containing protease inhibitors (Sigma, Shanghai, China). Protein samples were centrifuged and collected. The BCA method (Pierce, Rockford, IL, USA) was used to determine the concentration according to manufacturer’s protocol (Beyotime, Nanjing, Jiangsu, China). Subsequently, proteins as well as loading buffer were heated, after which proteins were separated by 10% SDS electrophoresis and transferred to polyvinylidene fluoride membranes (GVS Technology Co., Ltd., Suzhou, China). After blocked with milk at room temperature for one hour, membranes were incubated in a 1:1000 solution of primary antibodies (MST, MST2, LATS1, LATS2, YAP1, TAZ, TEAD1, TEAD2, TEDA3, TEAD4, GAPDH; Abcam, Cambridge, MA, USA) at 4 °C overnight. Subsequently, membranes were incubated with the secondary antibody at room temperature for one hour and observed using the ECL method (Pierce, Rockford, IL, USA).

### Statistical analyses

SPSS 19.0 (SPSS Incorporation, Chicago, IL, USA) was used for statistical analysis. Data were presented as mean ± standard deviation (SD). Comparisons among groups were analyzed by student’s t-test or one-way analysis. *P* < 0.05 was considered statistically significant.

## Results

### Verteporfin improves the effect of TACE in alleviating transplanted HCC

**Fig. 1:**
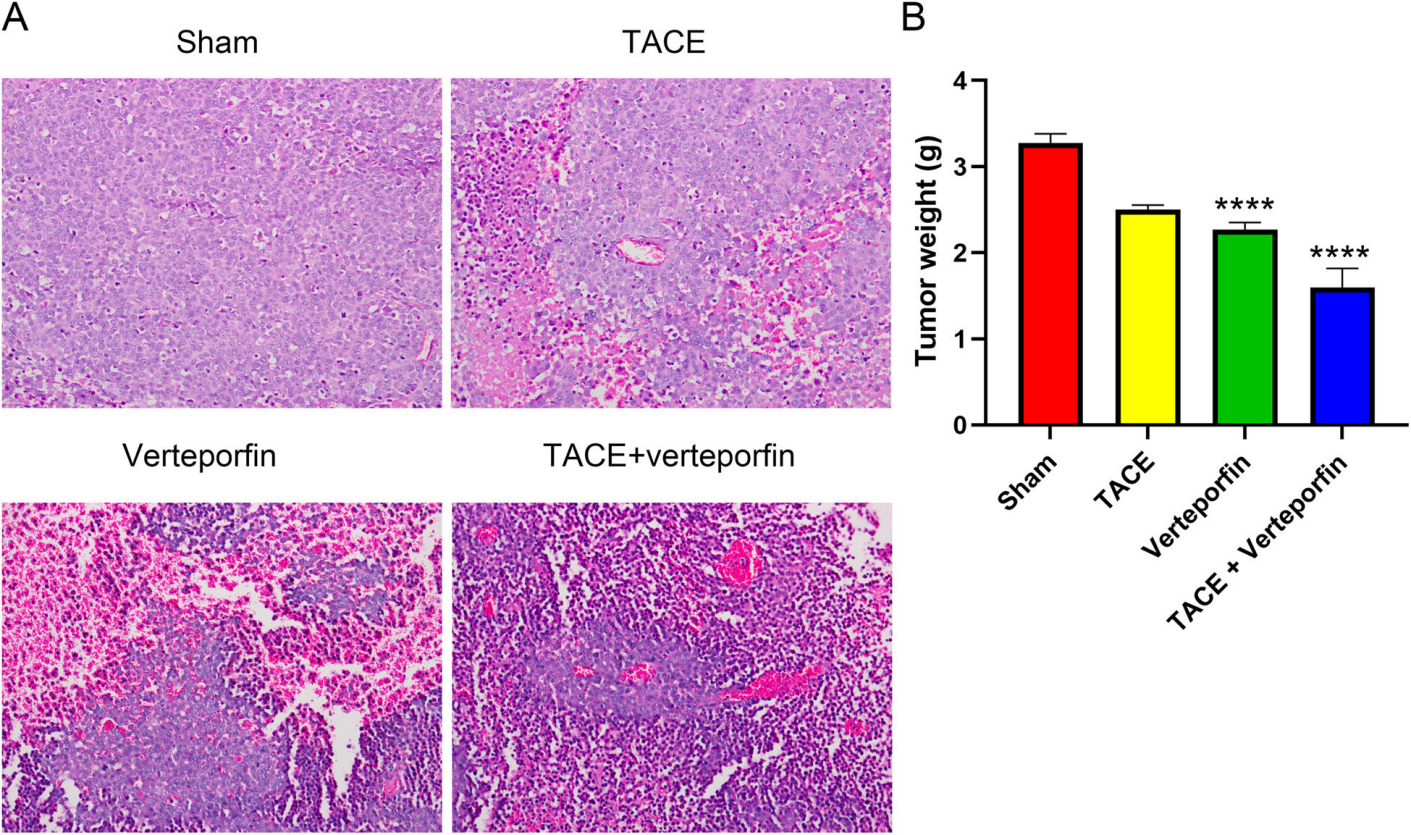
TACE combined verteporfin reduces the tumor weight of transplanted HCC. Verteporfin improves the effect of TACE in alleviating transplanted HCC. **A**, Morphology of tumor lesion in different groups. Morphology analysis showed that tissue injuries in the sham, TACE, verteporfin, and TACE+verteporfin groups were gradually lessened. Symptoms in the TACE+verteporfin group was significantly better than those in the TACE group (black arrow: normal tissue, the number of rats showing similar histopathology is 5); **B**, Tumor weight in different groups. And the tumor weight / body weight rates for 4 groups were 0.0132, 0.1040, 0.0088, 0.0064, respectively. (Results are presented as the mean ± SD, *n* = 5 per group; Compared with the Sham group, ****P < 0.0001; Compared with the TACE group, ^###^*P* < 0.001 and ^####^*P* < 0.001)

After the transplanted HCC model was constructed, tumor weight of transplanted HCC was measured. The tumor weights and tumor weight/body weight ratio in the TACE, verteporfin, and TACE+verteporfin groups were markedly reduced compared with the Sham group (*P* < 0.05) (Fig. 1B). Meanwhile, the tumor weight of the TACE+verteporfin group was significantly lower than that of the TACE group (*P* < 0.05). The results of the vehicle group revealed that dimethyl sulfoxide used in the present study had no significant effect on tumor or body weight. Morphological analysis showed that tissue injuries in the TACE, verteporfin, and TACE+verteporfin groups were alleviated compared with the Sham group and the symptoms in the TACE+verteporfin group was significantly better than those in the TACE group (Fig. 1A). These results showed that TACE combined verteporfin significantly suppressed the development of transplanted HCC.

### Verteporfin improves survival of rats after TACE therapy

**Fig. 2:**
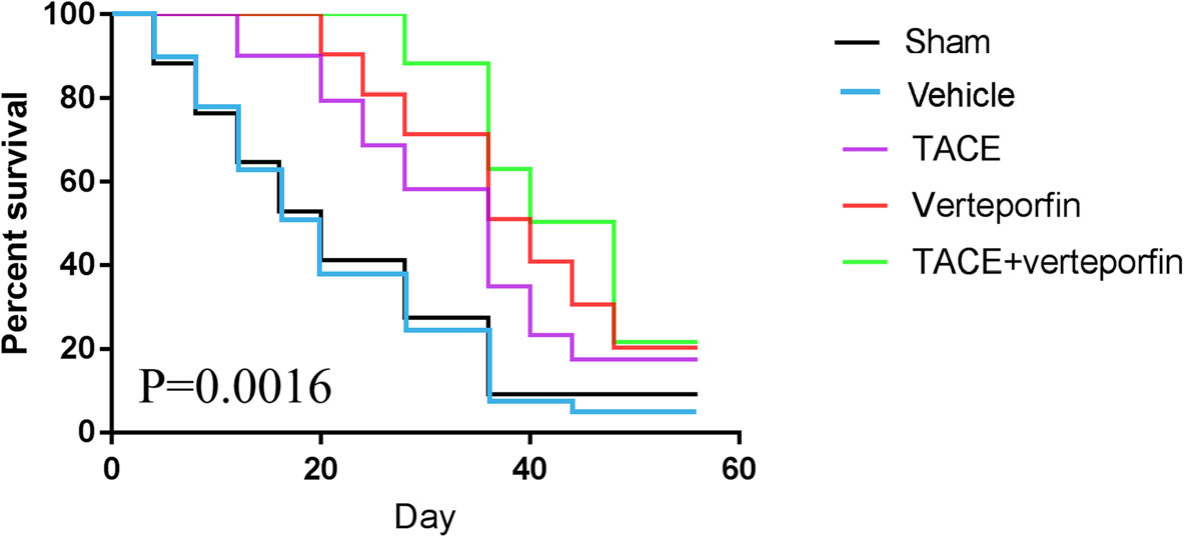
Overall survival analysis for transplanted HCC rats in different groups. Verteporfin improves survival of rats after TACE therapy. The overall survival (OS) of rats in different groups were analyzed by Kaplan-Meier curve. (Results are presented as the mean ± SD, *n* = 20 per group)

To further investigate the efficacy of TACE+verteporfin in treating transplanted HCC, the overall survival (OS) of rats in different groups was analyzed using Kaplan-Meier curve (survival endpoint was measured on the death of rats). The median survival time of the Sham, TACE, verteporfin, and TACE+verteporfin groups were 20d, 36d, 40d, and 48d, respectively. The OS rate of the Sham group was dramatically lower than that in the TACE, verteporfin, and TACE+verteporfin groups (*P* < 0.05). The TACE+verteporfin group showed significantly higher OS rate than that of the TACE group (*P* < 0.05) (Fig. 2). No significant difference was observed in the survival rate between the vehicle and Sham groups. These findings suggested that inhibiting the YAP signaling significantly improved the prognosis of HCC after TACE treatment.

### TACE plus verteporfin treatment could alter the mRNA expression effectors in hippo/YAP signaling pathway

**Fig. 3:**
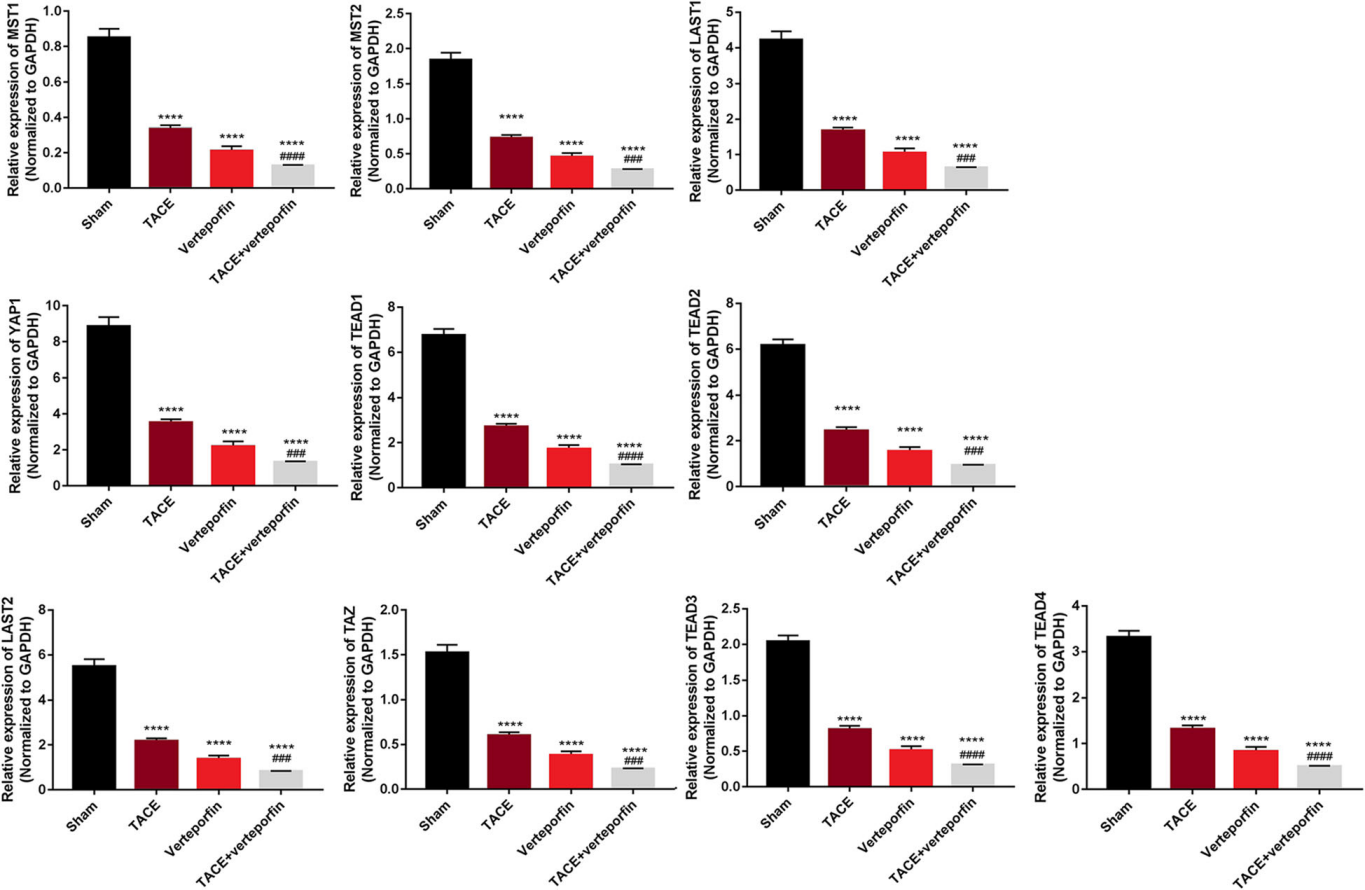
mRNA expression of effectors in Hippo/YAP signaling pathway in transplanted HCC rats. TACE plus verteporfin treatment could alter the mRNA expression effectors in Hippo/YAP signaling pathway. The mRNA expression levels of effectors in Hippo/YAP signaling pathway were determined by RT-qPCR. (Results are presented as the mean ± SD per group; Compared with the Sham group, ****P < 0.0001. Compared with the TACE group, ^###^*P* < 0.001 and ^####^*P* < 0.001)

To confirm the involvement of the Hippo/YAP signaling pathway, the mRNA expression of the effectors in the Hippo/YAP signaling pathway was determined by RT-qPCR. Both TACE and verteporfin significantly decreased the mRNA expressions of MST1, MST2, LAST1, LAST2, TAZ, YAP, TEAD1, TEAD2, TEAD3, and TEAD4 compared with the Sham group (*P* < 0.001). Moreover, TACE plus verteporfin significantly enhanced the downregulation of MST1, MST2, LAST1, LAST2, TAZ, YAP, TEAD1, TEAD2, TEAD3, and TEAD4 compared with the TACE group (Fig. 3, *P* < 0.001). These findings suggested that TACE plus verteporfin significantly inhibited the YAP signaling in transplanted HCC.

### TACE plus verteporfin treatment could alter the protein expression effectors in hippo/YAP signaling pathway

**Fig. 4:**
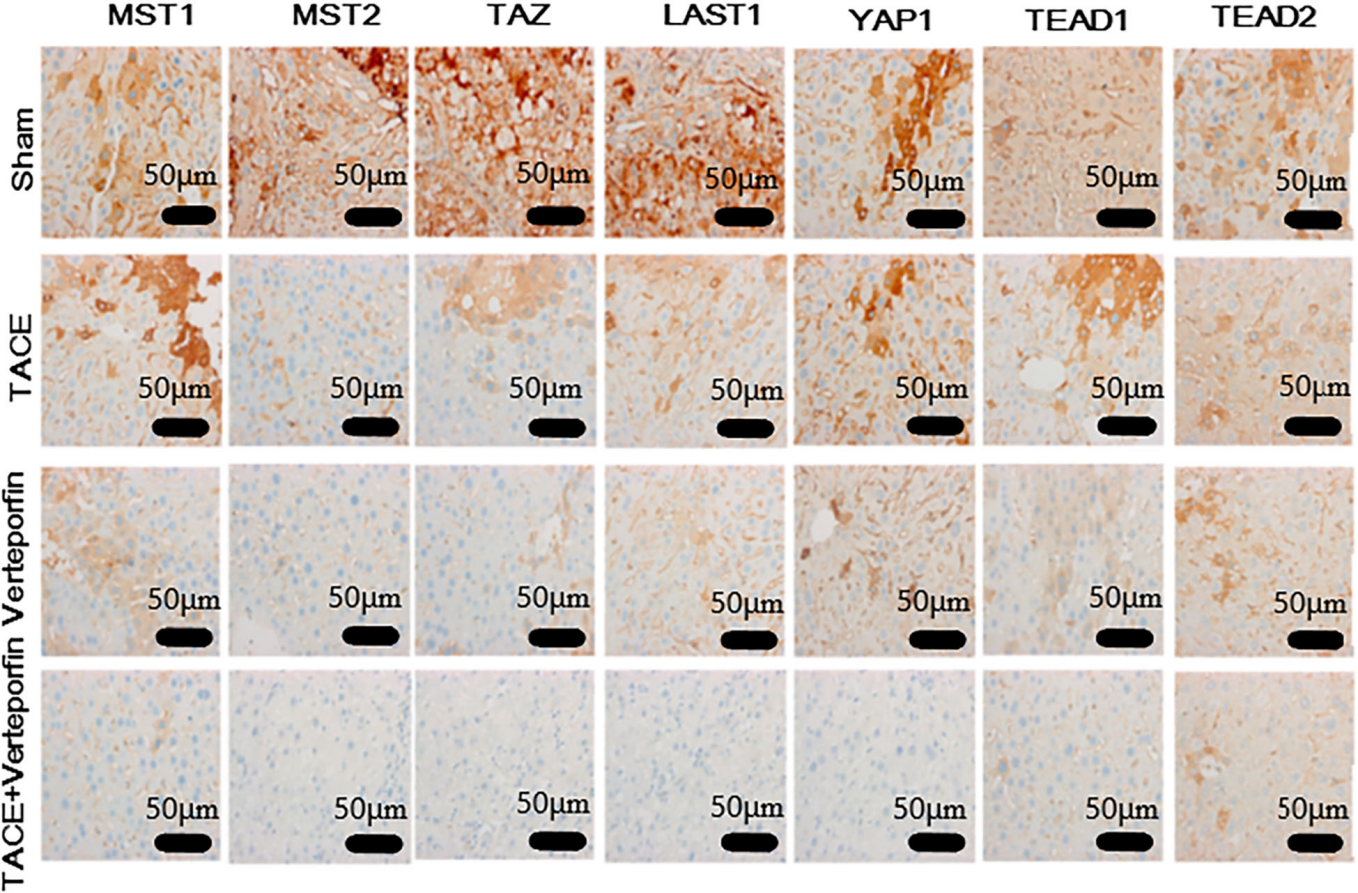
IHC detected expression of effectors in Hippo/YAP signaling pathway in transplanted HCC rats. TACE plus verteporfin treatment could alter the expression effectors in Hippo/YAP signaling pathway. The expression levels of effectors in Hippo/YAP signaling pathway were determined by IHC. Tumor tissues were used to observe the protein expression levels. IHC, immunohistochemistry

IHC was also performed to determine the expression of effectors in the Hippo/YAP signaling pathway. The result demonstrated that TACE and verteporfin significantly decreased the protein expressions of MST1, MST2, LAST1, TAZ, YAP, TEAD1, TEAD2, TEAD3, and TEAD4. TACE plus verteporfin significantly enhanced the expression of these proteins (Fig. 4).

### Overexpression of hippo/YAP signaling pathway reversed the effects of TACE plus verteporfin treatment

**Fig. 5:**
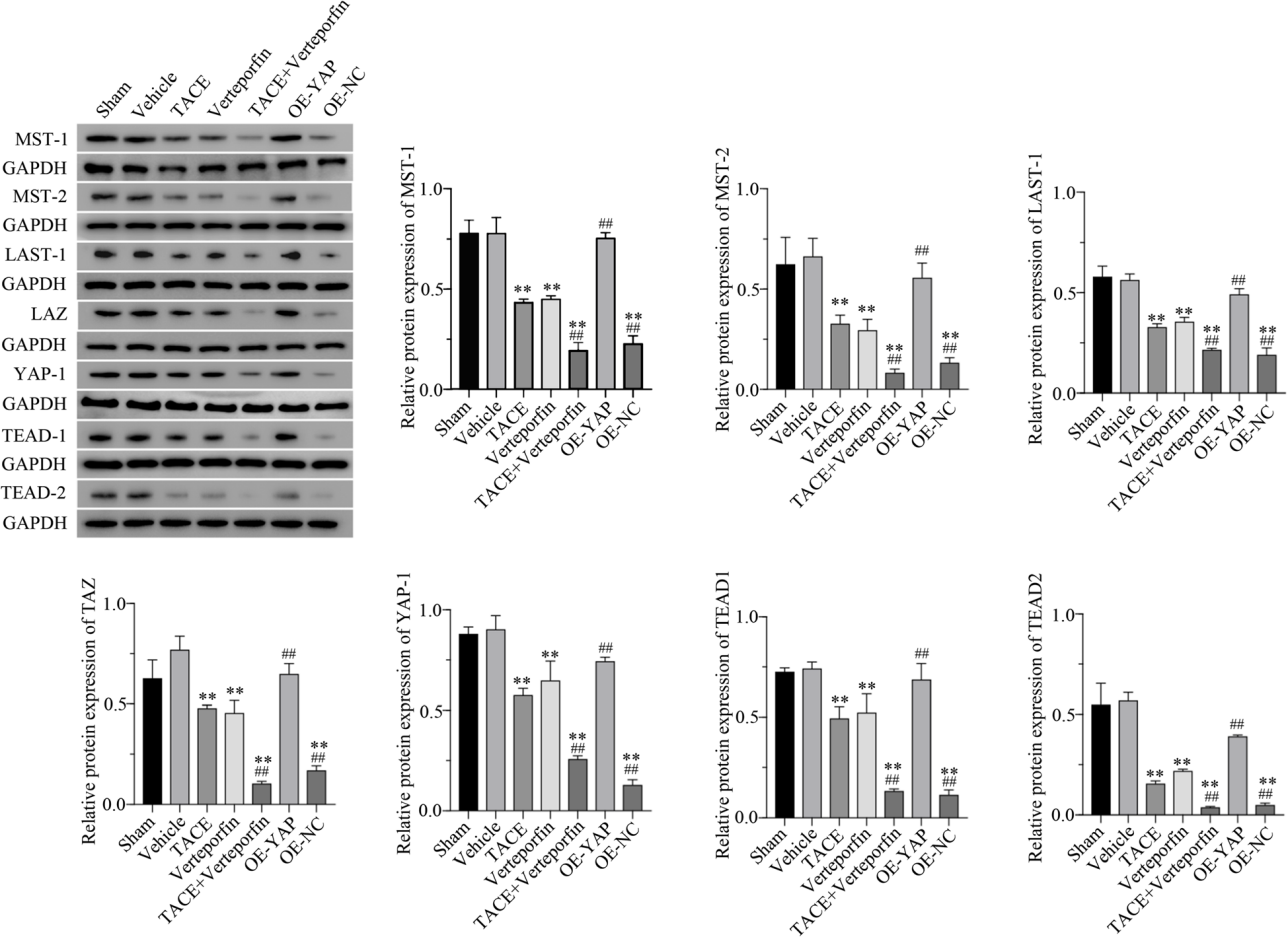
Western blotting detected protein expression of effectors in Hippo/YAP signaling pathway in transplanted HCC rats. TACE plus verteporfin treatment could alter the protein expression effectors in Hippo/YAP signaling pathway, while overexpression of YAP had reversed effects. The protein expression levels of effectors in Hippo/YAP signaling pathway were determined by western blotting. Compared with the Sham group, **P < 0.01. Compared with the TACE group, ^##^*P* < 0.01

**Fig. 6:**
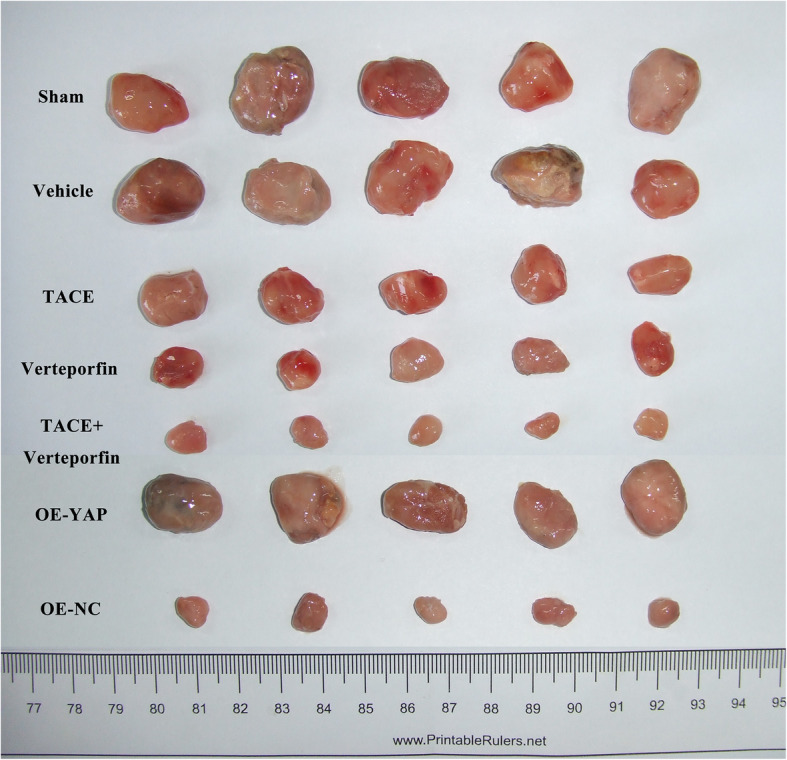
The tumor size of transplanted HCC. The tumor size was detected in the sham, vehicle, TACE, verteporfin, TACE+verteporfin, OE-YAP and OE-NC groups. TACE plus verteporfin treatment could decrease the tumor size, while overexpression of YAP increased tumor size

Furthermore, the protein levels of effectors in the Hippo/YAP signaling pathway were detected by Western blotting and IHC. Western blotting analysis suggested that both TACE and verteporfin significantly decreased the expressions of MST1, MST2, LAST1, TAZ, YAP, TEAD1, and TEAD2, and TACE plus verteporfin significantly enhanced these reductions (Fig. 5). However, the overexpression of YAP reversed the downregulation of these proteins compared to the OE-NC group. As shown in Fig. 6, the tumor size was decreased after treatment with TACE or verteporfin or both, while the overexpression of YAP resulted in the increase in the tumor size similar to the Sham group. The vehicle group showed that dimethyl sulfoxide had no significant effect on neither gene expression nor tumor growth. These data suggested that the overexpression of the Hippo/YAP signaling pathway reversed the effects of TACE plus verteporfin treatment.

## Discussion

HCC is one of the most common cancers and the third leading cause of tumor-related mortality worldwide. HCC-associated death is increased significantly in recent years [28]. Despite the efforts that have been made in recent decades, the outcomes and prognosis of patients with HCC remain dismal. TACE is a common therapeutic method for patients with intermediate-stage HCC and is of vital importance in alleviate the pathological condition of HCC patients [29]. However, there are still no global accepted guidelines for TACE treatment and the clinical outcomes vary among different individuals [30]. A study has demonstrated that sorafenib combined TACE treatment significantly improved the prognosis of patients with HCC [31], providing us a new insight in treating HCC. However, Lencioni et al. revealed that sorafenib combined TACE with doxorubicin-eluting beads did not improve the time-to-tumor progression compared with TACE with doxorubicin-eluting beads in clinics [32]. Thus, further investigation is needed to confirm these findings.

Previous studies have reported that the Hippo/YAP signaling pathway plays a crucial role in the development of HCC [33]. Verteporfin has been reported to be an inhibitor of the Hippo/YAP signaling pathway. In this study, the effect of inhibiting the Hippo/YAP signaling pathway on the outcomes of TACE in treating transplanted HCC was explored. The results showed that both TACE and verteporfin treatment reduced tumor weight and lesion and improved the OS ratio of transplanted HCC, indicating that the Hippo/YAP signaling pathway might have a significant impact on the pathogenesis of transplanted HCC. Interestingly, TACE combined verteporfin significantly improved the outcomes and prognosis of transplanted HCC compared with the TACE group, suggested that inhibiting the Hippo/YAP signaling pathway could significantly improve the outcomes of TACE in treating transplanted HCC. Previous study has revealed that YAP led to chromosomal instability in liver cancer [34], which might explain that inhibiting the Hippo/YAP signaling pathway improved the outcomes of TACE in treating transplanted HCC.

The effectors of the Hippo/YAP signaling pathway were detected in each group. Both TACE and verteporfin significantly decreased the expressions of MST1, MST2, LAST1, LAST2, TAZ, YAP, TEAD1, TEAD2, TEAD3, and TEAD4 [35, 36] compared with the Sham group. TACE plus verteporfin significantly enhanced these reductions. Thus, a combination of verteporfin and TACE suppressed the Hippo/YAP pathway by downregulating the expressions of these effectors. Previous study showed that LAST2 mediated the phosphorylation of YAP to regulate the pathogenesis of HCC [37]. Zhang et al. revealed that YAP interacted with HIF-1a to promote the stability of HIF-1a and induced cell glycolysis under hypoxic stress, indicating that inhibiting YAP expression could suppress the glycolysis of tumor, the energy provider for HCC [38]. Integrin α2β1 inhibits the phosphorylation of MST1 and activates the YAP signaling to promote the pathogenesis of HCC [39]. A recent study has also revealed that YAP/TAZ could be used as an diagnostic indicator for HCC [40]. In addition, NUP37 positively regulated the YAP/TEAD signaling to promote the development of HCC [41]. These findings suggested that TACE combined verteporfin significantly suppressed the development of transplanted HCC by inhibiting the expression of effectors in the Hippo/YAP signaling pathway.

### Study strength and limitations

The novel aspect of the current study was that the effect of inhibiting the Hippo/YAP signaling pathway in TACE-treated transplanted HCC was explored in vivo. The results highlighted the critical role of the Hippo/YAP signaling in the prognosis of TACE-treated transplanted HCC. The limitations of this study should also be addressed. First, the potential mechanism by which TACE plus verteporfin influences the Hippo/YAP pathway remains to be investigated. Second, other pathways independent of Hippo/YAP-associated factors were not taken into account; therefore, whether verteporfin affects other pathways is still unclear. Third, the effect of TACE combined with verteporfin on other organs is unknown.

## Conclusion

This study shows that the Hippo/YAP signaling pathway plays a critical role in the development of transplanted HCC. The inhibition of the Hippo/YAP signaling pathway by its inhibitor significantly improved the outcomes of TACE in treating transplanted HCC. These results suggest that TACE combined with verteporfin is a potential therapeutic method for HCC treatment.

## Supplementary Information

**Additional file 1: Figure S1.** Validation of the specificity of the antibody in IHC. The expression levels of effectors in Hippo/YAP signaling pathway in normal liver tissues vs liver tumor tissues were determined by IHC.

## Data Availability

The data are free access to available upon request.
