# Supplementary figures and images for: Transcatheter arterial chemoembolization combined with Hippo/YAP inhibition significantly improve the survival of rats with transplanted hepatocellular carcinoma
Source: Lipids Health Dis. 2021 Jul 25;20:74. doi: 10.1186/s12944-021-01486-w (PMC8310593; doi:10.1186/s12944-021-01486-w)

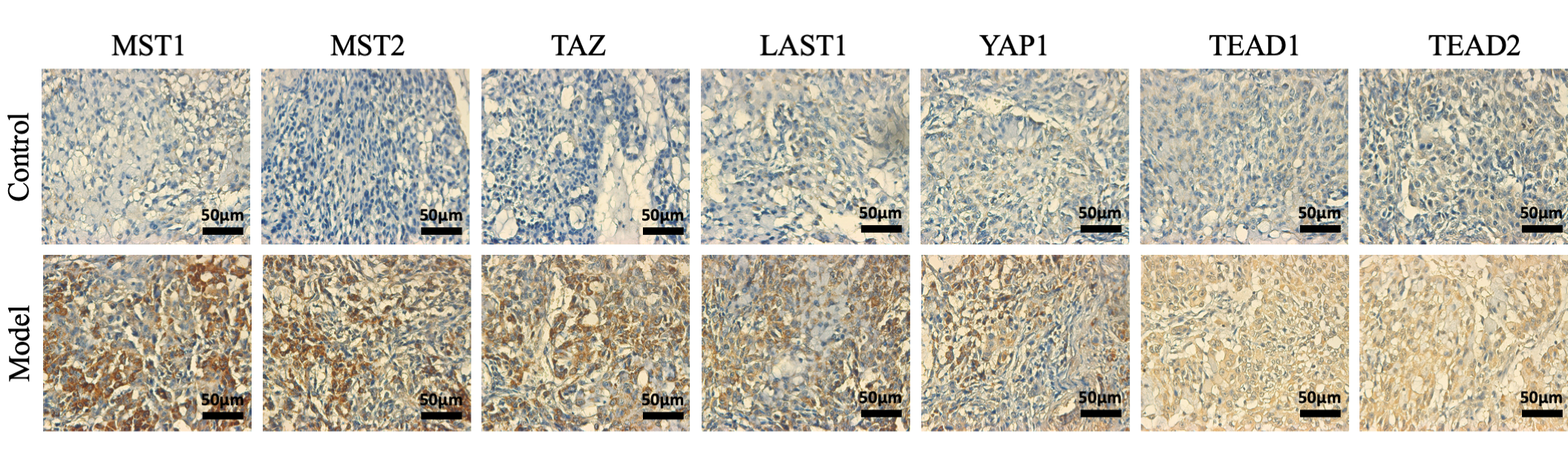

Supplement: Supplementary file 1 — Additional file 1: Figure S1. Validation of the specificity of the antibody in IHC. The expression levels of effectors in Hippo/YAP signaling pathway in normal liver tissues vs liver tumor tissues were determined by IHC. [file 12944_2021_1486_MOESM1_ESM.tiff]
